# Adapting the depression component of WHO Mental Health Gap Intervention Guide (mhGAP-IG.v2) for primary care in Shenzhen, China: a DELPHI study

**DOI:** 10.1186/s13033-022-00523-0

**Published:** 2022-02-15

**Authors:** Kendall Searle, Grant Blashki, Ritsuko Kakuma, Hui Yang, Shurong Lu, Baoqi Li, Yingying Xiao, Harry Minas

**Affiliations:** 1 Global and Cultural Mental Health Unit, Centre for Mental Health, School of Population and Global Health, University of Melbourne, Parkville, VIC 3010 Australia; 2 Nossal Institute for Global Health, University of Melbourne, Melbourne, VIC 3010 Australia; 3 London School of Hygiene and Tropical Medicine, London, WC1E 7HTE England, UK; 4 Monash Institute for Health and Clinical Education, School of Primary Health Care, Monash University, Notting Hill, VIC 3168 Australia; 5 Shenzhen Guangming Hospital of the University of Chinese Academy of Sciences, Bao’an District, Shenzhen, 518107 China

**Keywords:** Depressive disorder, Mental health gap intervention guide (mhGAP-IG.v2), Delphi, Adaptation, Conceptualisation, Primary care, Intersectorial care, World Health Organization (WHO), China, Shenzhen

## Abstract

**Background:**

Primary care doctors in Shenzhen, China are increasingly expected to identify and prevent depressive disorder; however, they have received limited mental health training and community healthcare centres (CHC) do not provide standardised protocols for the diagnosis and care of depressive disorder. The World Health Organization’s mental health gap intervention guide, version 2 (mhGAP-IG.v2) is a decision support tool for non-specialists for the assessment, management and follow-up of mental, neurological and substance use disorders (including depressive disorder). Given that mhGAP-IG.v2 is a generic tool, it requires adaptation to take account of cultural differences in depression presentation and unique characteristics of China’s emergent mental health system.

**Methods:**

A two-round, web-based, Delphi survey was conducted. A panel of primary care doctors from Shenzhen, were invited to score their level of agreement with 199 statements (arranged across 10 domains) proposing changes to the content and structure of mhGAP-IG.v2 for use in Shenzhen. Consensus was predefined as 80% panelists providing a rating of either “somewhat agree/definitely agree”, or “definitely disagree/somewhat disagree” on a five-point scale for agreement.

**Results:**

79% of statements received consensus with a mean score of 4.26 (i.e. “somewhat agree”). Agreed adaptations for mhGAP-IG.v2 included:- an assessment approach which considers a broader spectrum of depression symptoms and reflects the life course of disease; incorporating guidance for screening tool usage; clarifying physicians’ roles and including referral pathways for intersectorial care with strong family involvement; aligning drug treatment with national formularies; stronger emphasis of suicide prevention throughout all sections of the guide; contextualizing health education; reflecting a person-centred approach to care. Panelists chose to maintain diagnostic and treatment advice for bipolar patients experiencing a depressive episode as in the current guide.

**Conclusions:**

An adapted mhGAP-IG.v2 for depression recognises China’s cultural and contextual needs for assessment guidance; unique primary healthcare system organization, priorities and treatment availability; and diverse psychosocial educational needs. An adapted mhGAP-IG.v2 could both inform the future training programs for primary care in Shenzhen and also offer an additional mental health resource for non-specialists in other countries.

**Supplementary Information:**

The online version contains supplementary material available at 10.1186/s13033-022-00523-0.

## Background

The Healthy China 2030 Plan [1] outlines China’s 21st Century vision for healthcare and creates a favorable policy environment for mental health system reform of both national and global significance [2, 3]. At its core, it acknowledges the symbiotic relationship between a healthy working population and economic success [4]. It embodies key values of the United Nations’ Sustainability agenda [2, 5] and aligns with the latest global thinking on “person-centered, integrated care” [6–9]. Importantly, for a country with a sizable mental health treatment gap, where the vast majority of those with an identifiable mental health condition do not receive care [10, 11], the plan prioritizes the need to improve mental health awareness and prevention along with ready access to mental health services [12].

Shenzhen is a thriving metropolitan city located in Guangdong Province of China, just across the causeway from Hong Kong. Its population has rapidly expanded from 10.42 million people in 2010 to 17.56 million in 2020 with an average annual growth rate of 5.36% [13]. It was one of China’s first special economic zones and is nationally renowned for its progressive socioeconomic reforms [14, 15] which has transformed the region into a global commercial hub. Aware of increasing rates of mental disorders overall [16–18] and above national levels of prevalence for depressive disorder in key working and vulnerable populations [17, 19–23] (i.e. above 7.4%) [24], the local health authority committed to improving the recognition and prevention of common mental disorders within its developing primary healthcare sector[25]. Importantly, they invested in the upskilling of the GP workforce [26–28] and sought to implement the service of at least one doctor with a mental health certification in each community health centre (CHC) by 2020 [29]. Findings from qualitative research conducted with primary healthcare leaders in Shenzhen [30], however, found that along with improved professional development, doctors at CHCs also needed better access to diagnostic guidelines and protocols for depression care.

Guidelines can be considered as a “synthesis of evidence” that have been shaped into “practice-oriented recommendations” and represent the “highest quality opportunity” for patient care [31]. The sharing of guideline information, from one setting to another, avoids duplication of research efforts and optimizes on resource in underfunded settings [32, 33] and, as such, has become a widely accepted approach for global progress in healthcare [34]. One such guideline, the World Health Organization (WHO), Mental Health Gap Intervention Guide (mhGAP-IG) [35] offers community health care centers in Shenzhen a rigorously developed and internationally approved resource [36, 37] with which to train doctors on the assessment, management and follow-up of depression patients and other priority mental health conditions [38, 39]. Now freely available in its second edition (although not in Mandarin Chinese), mhGAP-IG.v2 [40] incorporates both evidence-based and financially affordable treatment options suitable for use in community and resource-poor settings.

mhGAP-IG.v2, however, is a generic tool and adoption needs to be accompanied by prior consideration of its context of use: “the physical, organizational, institutional and legislative structures that enable and constrain, resource and realize, people and procedures” [41, 42]. Whilst a universal approach to the adaptation of mhGAP-IG is still evolving [43], it is generally agreed upon that the consultation of end-users is integral for the appropriate adaptation and enhancement of future implementation [38, 44, 45]. These pre-requisites are particularly relevant to China, where substantive organizational and operational change to the national healthcare system have produced a model of primary healthcare delivery unique to its developmental context.

For example, mhGAP-IG embodies aspirational global standards in mental health practice which have been shaped by an established acceptance and confidence for the close involvement of primary healthcare in the diagnosis and management of mental health conditions in upper income countries [7, 46–48]. In contrast, primary healthcare doctors in China administer care without the legal authority to diagnose severe mental health conditions [30, 49]. Neither do CHCs experience the operational autonomy of western clinics. Instead, they are tied into the management system of their district hospital which controls both their budget and drug formulary [50]. Whilst Chinese psychiatrists were involved in the drafting of the initial mhGAP-IG, primary care representatives were not included in the consultation process and the guide may omit vital procedural or referral information in keeping with the realities of primary care prescribing practice.

Nor can the impact of culture on how different countries perceive, realise and respond to mental disorders at both a personal and system level, be ignored [51]. Socio-political events in China’s past have conditioned current-day attitudes and behaviors that can act as barriers to the expression of, and health seeking for, depression [52–54]. mhGAP-IG.v2 is closely aligned to international classification systems (e.g. ICD 10, DSM-IV) which were originally developed according to western presentation of disease in hospital settings and may not be sensitive enough to pick up cultural subtleties of disease presentation in community-based settings.

Whilst over 90 countries have seen the uptake of mhGAP-IG [39], publications focusing on its adaptation for country-specific needs are in short supply. The Programme for Improving Mental Health Care (PRIME) initiated investigations into the use, adaptation and integration of mhGAP-IG resources into the primary care systems across low and middle income countries [55, 56]. Since then, researchers based in Africa [38, 57–59] and Nepal [45] have endeavored to either contextualize or provide a culturally sensitive translation [59] of the guide. This was commonly achieved by engaging key stakeholders in a multi-stepped review, generally led by mental health specialists or a discrete research team [38, 57, 58] and including Primary care representatives in at least one stage of the adaptation process (i.e. as participants of preliminary qualitative focus groups [38, 57] for prototype testing of a training program [58].

This research uses the Delphi Approach, an established consensus methodology (see methods section for more details), to adapt the depression component of the mhGAP-IG.v2 for use by doctors working in community healthcare centres in Shenzhen, to take into account local culture and health system structure and reform. It signifies the completion of three phases of research, all of which have been conducted “*with primary care, for primary care*”: from its conception with primary care leaders to explore the current needs for the improved management of depressive disorder in CHCs [30]; to immersive workshops with primary care leaders to compare the depression guidelines with current conceptualisations of depression and clinical practice (awaiting publication); through to this Delphi study with the broader primary care community in Shenzhen.

It is unique amongst these adaptation studies by its intentional focus on the depression component only (i.e. one chapter of the guide). With the exception of one study in Nepal [45], which focused on dementia, other key studies in this field were directed at reviewing the whole guide [38, 57–59]. The advantage of this depression-only approach is the breadth and depth of items, generated from highly focused preliminary research that can be put forward for consensus testing. As such, there is potential to obtain the systematic consensus on guideline adaptations across a range of topical mental health system, depression symptom presentation and other complex care-related concepts, which have not been previously explored in this way. Importantly, it seeks to support local and global aspirations of reducing the mental health treatment gap in China.

In summary, this study occupies a vital bridge between international guidance on best practice for depression care and making knowledge relevant and acceptable for the end-user of the guide. To the best of our knowledge, it is the only mhGAP-IG.v2 adaptation study initiated in China within the primary healthcare sector.

## Methodology

### The Delphi approach

Delphi is a systematic review process that obtains a consensus view on a matter from a panel of experts. An expert can be anyone who has intimate knowledge, practice, experience or need relating to the topic of interest. The panel need not be very large [60] nor need to establish statistical power [61] but key conditions must be met, including diversity of expertise; independent and decentralised decision making; and aggregation of results by an objective research facilitator [62]. Delphi has been widely used by the mental health sector and often when other sources of evidence is unavailable [63]; for example, to define standards of practice for a mental health workforce [64] or to assist with the content development of mental health training programs [65, 66]. This research applied the Delphi approach to establish a primary care panel to participate in two rounds of a web-based, self-completion survey (in Mandarin Chinese). Panelists were asked to review and score their level of agreement with a series of statements proposing adaptations to the content and structure of the mhGAP-IG.v2 to contextualise it for use in China.

### Panel recruitment

A Shenzhen-based steering committee of seven Primary Healthcare Leaders (two from the core urban area; four from the suburban areas; one from the city border districts) facilitated recruitment and provided advice on local conditions. Leaders had been identified by their prior graduation from *The Shenzhen General Practice Leadership Training Programme *[67]*,* an international educational collaboration between Monash University, Australia, and the Shenzhen Health Capacity Building and Continuing Education Centre, the regulatory body and vocational training provider responsible for the qualification and certification of primary care doctors in Shenzhen. They circulated survey information through their district primary healthcare network, distributed and collected the plain language statements and informed consent forms in accordance with ethics requirements. The research co-ordinator (KAS) finalised enrolment by providing each panelist with a confidential and password-protected link to the live online survey. She was also responsible for intra-wave analysis and feedback to panel members.

### Inclusion/exclusion criteria

Doctors working in CHC who saw 50 + patients each week were invited to join the panel. Doctors required either a self-professed interest in mental health and/or a primary healthcare mental health certificate (i.e. did not need to be actively diagnosing depression patients as this function is considered to be the role of specialists in China). Survey materials were provided in Mandarin Chinese; thus English was not a pre-requisite. Panel heterogeneity was achieved by recruiting doctors working in CHCs located across urban, suburban and city boarder areas of Shenzhen.

### Size and geographic distribution

75 doctors (51% Female) participated in the first wave of the survey with most panelists working in CHCs located in suburban areas (55%), followed by core urban districts (35%), then by city border districts (11%), where international academic relationships are least well developed. Fewer panelists, 59 in total, participated in Wave 2 (retention rate, 79%). Despite the dropout, the overall percentage contribution by area and gender remained similar across both waves of the survey. Doctors with a mental health certificate were more likely to participate in both waves of the survey.

### Questionnaire development

The statements were generated through the thematic analysis of bilingual qualitative research regarding the acceptability, applicability and transferability of mhGAP-IG.v2 use in CHCs as conducted previously by this group with primary care leaders (awaiting publication). The statements were organized into ten domains relating to: (i) person-centred concepts of care; (ii) depression presentation; (iii) screening instruments; (iv) healthcare interconnectivity; (v) drug treatment; (vi) mania; (vii) communicating depression; (viii) follow-up; (ix) managing patient information; and (x) overall guide structure. Statements were translated into Mandarin, back-translated, piloted (3 CHC doctors in Shenzhen and 2 GPs/researchers in Australia) and refined. Finally, 199 statements were put forward for testing in Wave 1.

### Questionnaire metrics

Statements were scored for agreement using a five-point Likert scale where 5 = “definitely agree”; 4 = “somewhat agree”; 3 = “neither agree or disagree”; 2 = “somewhat disagree”; 1 = “definitely disagree”. Consensus was pre-determined as 80% panelists scoring either a 4/5 (i.e. “somewhat agree/definitely agree”) or conversely 1/2 (i.e. “definitely disagree/somewhat disagree”) on the five-point Likert scale for agreement. Panel members also had the opportunity to suggest additional items to the guide or to express any reservations through some open-ended questions.

### Survey process

**Fig. 1:**
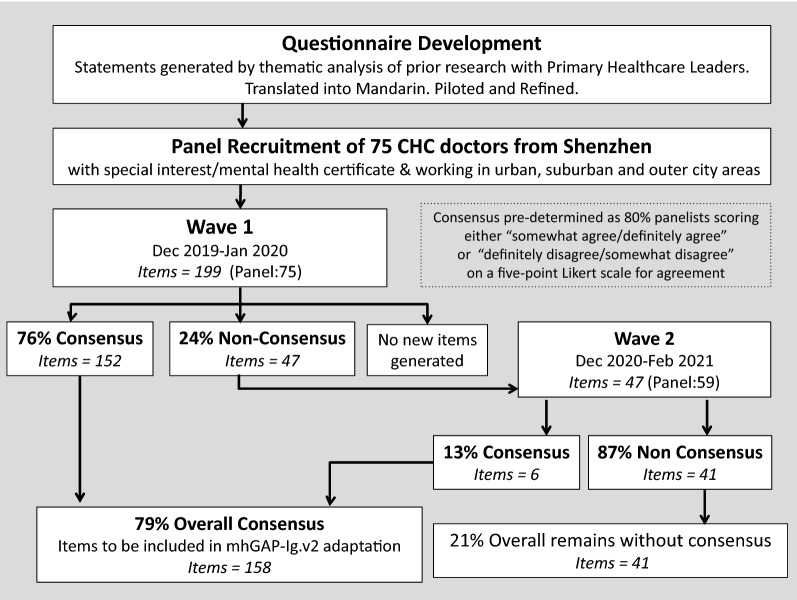
The Delphi process from questionnaire development to statement consensus

Two waves of web-based survey were conducted using the Qualtrics Survey Platform (Wave 1: December 2019 to January 2020; Wave 2: December 2020 to February 2021). In the first wave of the survey, 152 items (76%) met consensus. Open-ended responses were assessed and no new statements were generated. The remaining 47 statements, which did not reach consensus, were re-presented to the panel in a second wave of testing. To assist the decision making process, panelists were provided with the overall rating for each item as well as their individual rating compared with the rest of the panel. In the second wave of the survey, 6 more items received agreement bringing the final agreed number of items to 158 (accounting for 79% of all rated statements). After wave 2, doctors remained undecided on 41 items. Finally, recommendations for guideline adaptation were established by comparing consensus (and non-consensus) items against the relevant sections of the mhGAP-IG.v2. See Fig. 1.

## Results

### Demographics

*Panelist characteristics:* Overall, doctors had an average of 6 + years of formal general practice experience. In Wave 1, 15% of panelists personally held a mental health certificate and 57% had access to another GP with mental health certification within their CHC. In Wave 2, more panelists either held a mental health certificate (24%) or had access to another GP with mental health certification (68%). A sub-analysis confirmed that, during the course of the study, several panelists received mental health training and achieved certification as part of their health center’s commitment to “one mental health GP per centre”.

*CHC characteristics:* In general, panelists worked in a clinical team with an average of 7 to 8 doctors, although clinic size ranged widely from 1 to 20 doctors. Panelists in Wave 2 had a significantly lower patient load per week than those in Wave 1 (Wave 2: 176.3 patients/week versus Wave 1: 275 patients/week). Similarly, Wave 2 panelists also recorded fewer depression diagnoses (Wave 2: 1.9/week versus Wave 1: 2.7/week). A sub-analysis and follow-up telephone calls with several panel members confirmed a temporal change in health seeking behavior during the one-year interim between survey waves, which coincided with the Covid-19 epidemic. During this time, people were advised not to visit the doctor (unless in emergency) and doctors focused their time on the vaccine rollout.

### Consensus overview

Upon completion of research, Panelists “somewhat agreed” with 158/199 proposed adaptions to the mhGAP-IG (overall adjusted mean score: 4.26 up from 4.25 in Wave 1). The adjusted mean scores for each domain ranged from 4.05–4.65 with statements relating to “Developing a patient-centred guide” receiving the highest adjusted mean score and “Follow-up” the lowest. In Wave 1, domains for “Developing a patient-centred guide” and “Bipolar disorder” received 100% consensus on all items. Thus the 47 items put forward for Wave 2 came from eight domains only. Further consensus was limited, with only between 17–31% of items clustered within three domains receiving further agreement. The domain with the most intra-wave movement was “Follow-up” where the percentage consensus increased from 64 to 73% across the two waves of research. Finally, no items, from any domain, in either wave of research, were rejected outright (i.e. scored either 1 or 2 by 80% or more doctors). (See Table 1).

**Table 1:** Wave 1 & 2 Delphi Survey Consensus by Thematic Domains

	Wave 1 Base: 75 panelists			Wave 2 Base: 59 panelists			Overall Consensus	
DOMAIN	No. of Items Tested	Consensus %	Mean Score	No. of Items Tested	Consensus %	Mean Score	Consensus %	Adjusted Mean Score
Overall Consensus	199	76	4.25	47	13	3.66	79	4.26
1. Developing a Patient-Centred Guide	18	100	4.65	0	Na	Na	100	4.65
2. Symptom Presentation of Depression	28	64	4.07	10	0	3.50	64	4.06
3. Improving Access/Usage to Diagnostic Tools	11	64	4.13	4	25	3.80	73	4.24
4. Healthcare and Community Interconnectivity	28	79	4.23	6	17	3.75	82	4.26
5. Considering Pharmacological Interventions	18	78	4.20	4	0	3.51	78	4.19
6. Considering Mania and Depressive Episode in Bipolar Disorder	4	100	4.59	0	Na	Na	100	4.59
7. Communicating Depression to Patients and the Community	29	90	4.36	3	0	3.77	90	4.36
8. Follow-Up	33	61	4.03	13	31	3.64	73	4.05
9. Managing Patient Information	11	82	4.20	2	0	3.73	82	4.20
10. Overall Structure of an Adapted Guide	19	74	4.06	5	0	3.61	74	4.06

^*^^1^Consensus based on 80% panel participants providing a score of either 4 or 5 (i.e. “Somewhat agree/Definitely agree”) or conversely a score of 1 or 2 (i.e. “Definitely disagree/somewhat disagree”) for each individual item

^*^^2^Wave 2 items were drawn from all those items where consensus was not achieved in Wave 1. Additionally, no items were rejected in Wave 1 (i.e. 80% participants scoring either a 1 or a 2 (i.e. “Definitely disagree/Somewhat disagree”)

### Domain specific results

Table 2 outlines panelists’ recommendations for adaptations to mhGAP-IG.v2 based on statements that received consensus after two waves of Delphi survey. It is grouped by domain (ten in total) and listed according to decreasing mean score for overall domain agreement. Full listings of items, with and without consensus, are available as Additional file 1: Tables S3 and S4.

**Table 2:** Primary care recommendations for an adapted mhGAP-IG.v2

DOMAIN	Primary Care Recommendations for an Adapted mhGAP-IG.v2
Rank ordered by mean score	Based on all items receiving consensus after two waves of Delphi survey *(Wave 1: 75 CHC doctors; Wave 2: 59 CHC doctors)*
Developing a patient-centered guide	1.1 Not just patient-centred but person-centred Ensure patient privacy Sensitive to events in patients life (i.e. grief) Accommodate for patient diversity 1.2 Provide a personalised patient management plan (PMP) developed in partnership with the patient flexible to evolve over time to suit patient needs outlines several strategies to elicit positive behaviour change 1.3 PMP to incorporate doctor, patient and administrative outcomes:- Doctor review of treatment progress (e.g. drug side effects, treatment changes) patient’s treatment evaluation (e.g. treatment experience and engagement with therapy) administrative tracker (e.g. consultation appointments and potential referral options) 1.4 Doctor with mental health certification to develop PMP template 1.5Address contextual differences Consider suicide risk as a priority Highlight family involvement in every stage of care Provide specific examples of patient success stories from Shenzhen
Depressive episodes in bipolar disorder	2.1 Differentiate between treatment practice for (uncomplicated) depression and depressive episode in bipolar disorder mood stabilizer required as an adjunct to antidepressant treatment 2.2 Check for symptoms of mania with the patient or with family members
Communicating depression to patients and the community	3.1 Doctors to play a role in improving community awareness and health literacy 3.2 Stronger emphasis of the role of family in patient support 3.3 Include strategies for CHC to monitor patients at risk of suicide 3.4 Expand content of patient psychoeducation include approaches to develop patient trust (e.g. listening with empathy) address any misconceptions about the disease provide treatment success stories prepare patients for community stigma discuss the importance of treatment adherence provide advice on engagement with activities encourage attendance of follow-up appointments with CHC/specialists agree to a management/healthcare plan provide advice on self-care Consider implementing an appointment system
Healthcare interconnectivity	4.1 Highlight community involvement with monitoring patients at risk of suicide 4.2 Clarify referral pathways and the division of professional responsibilities 4.3 Highlight opportunities for medical and non-medical intersectorial care* 4.4 Reference involvement of family as a component of intersectorial care 4.5 Include a reminder to keep the patient central to all discussions
Access/usage to depression questionnaires	5.1 Recommend questionnaire usage during management and/or/both follow-up 5.2 Questionnaires conducted in a private consultation room 5.3 Doctors & nurses can administer questionnaire (assuming training provided) 5.4 Clarify scope for tools (i.e. multiple times? non-clinic settings?)
Managing patient information	6.1 Review a patient’s history before consultation and update patient records 6.2 Supplement information from patient management system if necessary 6.3 Validate patients’ treatment with specialists, other treating doctors, family members or by sighting medication 6.4 Hold regular in-clinic meetings to discuss difficult cases with other doctors
Considering pharmacological interventions for depression	7.1 Address Dr’s role with regards to monitoring and changing drug dose 7.2 Include indication and side effect profiles for each drug group 7.3 Include drug availability & reimbursement status for each drug group 7.4 Include adherence advice for each drug group 7.5 Provide details of latest generation of drugs 7.6 Provide Chinese brand names 7.7 Add information for Benzodiazepines
Symptom presentation of depression	8.1 Guide should “reflect a real-life consultation” consider whether a patient had had a previous episode of depression ascertain details of relationship problems (including grief) assess patient’s risk of suicide early in consultation consider physical symptoms first 8.2 Include triggers and risk factors for depression 8.3 Diagnosis based on “a symptom spectrum rather than core symptoms alone” merge both the core and additional symptoms into one listing include loss of libido in symptom listing order the list by good predictors of depression/commonly seen symptoms provide additional details of symptom profile changes over time 8.4 Structural clarity to differentiate between physical and mental symptoms 8.5 Additional symptom information required for children 8.6 Careful quantifications/translations required for “sleeping too much” “talking and moving more slowly than usual”
Overall structure	9.1 Some or major restructuring needed to better reflect the context of use 9.2 Emphasise intersectorial involvement including family/community network 9.3 Highlight differences between depression and a depressive episode in bipolar disorder 9.4 Integrate follow-up and management into one continuous section 9.5 Commence assessment with consideration of patients at high risk of suicide 9.6 Place the patient at the centre of the guide
Follow-up	10.1 Define follow-up and explain why it is necessary to determine a patient’s treatment status to establish previous therapy/treatments received to monitor current treatment side effects & assess for improvement 10.2 Explain how to assess for improvement:- i.e. itemise signs of improvement 10.3 Highlight key CHC outcomes to be achieved from follow-up consultation options for basic psychotherapy at CHC level referral options for either psychological interventions/drug treatment 10.4 Itemise modes of follow-up contact (face-to-face, telephone, electronic) 10.5 Recommend preferred mode of consultation contact ideally, face-to-face follow-up for all patients at least, face-to-face for all those with serious conditions patient’s preference for non-emergency cases 10.6 Frequency and duration of contact determined by depression severity, risk of self harm and treatment compliance

^*^schools; police; social services; work-place support groups; voluntary sector; other mental health support services; disabled persons federation

Of highest approval was developing a patient-centred guide. Panelists consented to an “in principal” person-centred approach to care with additional emphasis given to patient privacy, sensitivity towards patient diversity and personal events of significance. To achieve this, they supported the provision of a patient-led management plan and agreed it should include both patient-led outcomes, doctors’ clinical feedback and administrative milestones. As this is not current practice in Shenzhen, they allocated responsibility for template development to be held by the CHC doctor with mental health certification. They also universally agreed that guidelines should emphasize suicide as a priority concern in primary care and the active involvement of family in care. These two elements reflect unique differences between “norms” in China’s primary care health system and mhGAP-IG.v2.

Panelists unanimously consented to incorporating all information in mhGAP-IG.v2 relating to differential needs for the treatment of a depressive episode in bipolar disorder. Again, they highlighted the importance of involving family in the guidelines for care by actively asking for their input regarding mania symptoms.

To be more relevant for the Shenzhen context, panelists wanted to expand the mhGAP-IG.v2 scope to approve doctors’ role to educate patients, family and community members about depression as an important step to improving overall health literacy. Furthermore, they endorsed CHC doctors playing a leading role with monitoring suicide in the community supported by the inclusion of additional strategies. They consented to updating the section on psycho-education to further contextualise it for use in Shenzhen, with a particular need to address establishing patient trust. Panelists saw fit to use the guide to promote using an appointment system as a practical solution to improve access to doctor time.

Two key changes to mhGAP-IG.v2 were agreed upon to enhance healthcare interconnectivity and demonstrate a context-specific commitment to intersectorial care. Firstly, the flowcharts relating to referral options should encompass a wide range of medical and non-medical services (e.g. schools; police; social services; work-place support groups; voluntary organisations; other mental health support services; disabled persons federation*).* Secondly, family members need to be incorporated as key components of intersectorial care. Importantly, the schema of the guide should also project a sense of keeping the patient central to decision-making.

Panelists took the opportunity to expand the guidelines to provide advice on the potential use of depression screening tools to support a depression diagnosis (i.e. by whom; at what stage of care; how often; in what settings). Practical measures to improve the management of patient information were also approved, such as validating patient treatments directly with patient and/or treating doctors. Additionally, they endorsed the practice of case sharing with other doctors within the same clinic.

With regards to adapting the table of pharmacological treatments for depression, experts concurred that drug listings should provide Chinese brand names and include the latest generation products. As well as standard product indications and side effect profile information contained within the current guide, CHC doctors had additional information needs to cover product availability (CHC versus hospital), reimbursement status and compliance advice. Contrary to the current focus on antidepressants only, panelists conceded to introducing information on Benzodiazepines. Importantly, they agreed that drug information should be preceded with a clarification of the CHC doctor’s role in monitoring and changing antidepressant treatment.

Whilst Panelists struggled to come to agreement on all of the items relating to depression presentation, they consented to a conceptual change to diagnostic practice such that *diagnosis should be based on a spectrum of symptoms rather than core symptoms alone*. They endorsed a key structural change to the assessment section (i.e. merging core and additional symptoms into one listing). Whilst they did not concede to expanding the content of the merged listing to include anxiety and a range of depression-related behavioral symptoms (e.g.. weeping) (See additional files for non-consensus items), other agreements portrayed a preference to reflect a real life consultation and the use of risk factors to support recognition of depression. It was agreed that children would need additional information.

Several structural changes to the overall guide were recommended, particularly relating to the algorithms for follow-up practice, which do not currently resemble the CHC reality. Panelists approved statements to merge the management and follow-up sections of the current guide. Additional educational elements would need to be added to the new section: to define the purpose of follow-up; to explain how to assess for improvement; and to provide outcome measures such as context-specific referral options to care. Details relating to frequency, duration, and appropriate mode of follow-up contact were also needed. Panelists reinforced the need to bring suicide to the forefront of the guide and embed family as part of the cycle of care. Furthermore, any changes are not to deflect from the intrinsic principle of keeping patients central.

## Discussion

### Context-specific needs for assessment guidance

This research builds on findings from preliminary research conducted by this group with primary health care leaders in Shenzhen (awaiting publication), which confirmed CHC doctors’ preference to base a depression diagnosis on “a spectrum of symptoms, rather than core symptoms alone”. The agreed merger of both the core and additional symptoms into one listing in an adapted guide is symbolic of the pragmatic nature of primary care, which avoids the premature exclusion of a potential diagnosis in favor of ongoing monitoring and care. It signifies an opportunity to move away from the underlying, specialist-developed diagnostic criteria of mhGAP-IG.v2 to adopt a life course approach, befitting with primary care (i.e. that depression is a complex multi-dimensional disease where symptoms can present in any order over the course of a lifetime) [7, 68, 69]. Although panelists did not endorse primary healthcare leaders’ suggestions to incorporate somatic symptoms, anxiety and other behavioural indicators for depression into this listing*,* they agreed more generally that mhGAP-IG.v2 should inform doctors of potential risk factors which trigger depression and clarify how depression changes over time.

A key finding of this research was the consistent endorsement of modifications throughout the mhGAP-IG.v2 to reinforce primary care’s responsibility to detect patients at risk of suicide. In diagrammatic terms, this necessitates a reorganization of the decision-making flowcharts, to check prior history of suicide attempt early in consultation, to elevate suicidal tendencies in the symptom listing and to direct the doctor to the module for self-harm, also contained within mhGAP-IG.v2. These changes are both consistent with China’s national drive to reduce suicides by 30% by 2030 [70] and necessary to address locally-based concerns of high rates of suicides in key working and vulnerable populations [71, 72]. Similarly, self-harm and suicide was identified as a priority disorder in a contextualization study in Tunisia [58].

The current version of mhGAP-IG.v2 does not provide any guidelines regarding the use of depression screeners in the routine diagnosis of depression, despite the availability and clinical use of a range of GP validated depression questionnaires in the West [73]. Context-specific findings from previous research indicates that CHCs in Shenzhen do not have standard access to depression screeners [30]. Panelists saw an opportunity to extend the guidance to encourage the uptake of appropriate screening tools (assuming appropriate training) in Shenzhen. They recognized that there was potential to involve nurses in the assessment process and this should be highlighted in the mhGAP-IG. The openness to consent to the inclusion of these new items into the guide perhaps reflects CHCs’ contemporary task-shifting needs and willingness to find solutions to improve the detection of depression.

Panelists did not seek to modify all aspects of the mhGAP-IG.v2 as demonstrated by their universal adoption of all information relating to bipolar disorder. The prevalence of bipolar disorder is reported to be low in China [74]. Preliminary research suggests that it is poorly understood by primary care doctors and is categorized as a severe mental health condition requiring specialist treatment. Panelists respected the guide’s ability to fulfill a relevant knowledge gap in primary care training and chose to maintain recognition and treatment strategies to differentiate between a patient with depression and a patient with bipolar (experiencing a depressive episode).

### Responding to health system differences

This research augments findings from prior research conducted by this group with primary care leaders in Shenzhen and other contextualization studies to clarify within the guide country-specific referral options and the division of medical professionals’ responsibilities [38, 57, 58]. China’s national health system reform aspires to integrate mental healthcare into primary care [50, 75]. Key policy documents, however, do not provide step-by-step details on how to manage this transition [3, 76]. Whilst in many western countries, GPs have been actively involved in both the diagnosis and treatment of depression for some time [77, 78], these reforms represent a significant departure from current norms in China where psychiatrists are primarily responsible for diagnosis and treatment [49, 79]. The contextual updating of mhGAP-IG.v2 provides an opportunity to showcase how the newly developed role of CHC doctors with a certificate in mental health [29, 30] interfaces with current referral networks to provide the vital interconnection between the two arms of China’s evolving mental healthcare system (i.e. community and hospital medicine).

The depth of this study allows panelists to endorse guide content relating to emergent pathways of care – specifically those achieved by integration of primary care service with community bodies (e.g. social services, police, schools, voluntary sector, disabled persons federation), employers and family members. Panelists’ preference for a strong community and family focus has both a cultural and a legal precedent. In contrast to many Western countries, China’s mental health law stipulates that family members must be responsible for the care of family members with mental health concerns (i.e. helping to administer medication, ensuring the patient attends medical appointments, mood monitoring) [3, 49, 69, 76]. Indeed, other public health interventions (e.g. mental health first aid) also harness this dynamic to recognize and support depression cases in the community [80]. Clearly, the current management and follow-up sections of mhGAP-IG.v2, which place the doctor in control of disease management, are not a good match for a health system that devolves mental wellbeing to a wider range of community actors.

### Accounting for locally available drug treatments

As with other key mhGAP-IG contextualisation studies [38, 57], this research highlighted that antidepressant drug listings (e.g. mhGAP-IG.v2, Table 2, pg 29) need to be aligned with national formularies. In this case, where antidepressants are not standardly available in CHCs, information regarding the site of availability (i.e. hospital versus CHC) and drug-reimbursement status should be provided. Panelists also agreed to improve the currency of the guide by updating drug listings with the latest generation of selective serotonin reuptake inhibitors (SSRIs) and including brand names. Shenzhen is an affluent city, where disposable incomes can be very high [81], pharmaceutical drug representatives active and patients potentially aware of drug-options through direct-to-consumer marketing. However, including branded products within the mhGAP-IG.v2 may prove to be problematic, when guidance needs to maintain neutrality and avoid the promotion of one brand in preference to another. It also goes against local health authority thinking which aims to avoid over-prescription and patient addiction to potentially harmful drugs.

Panelists were also in consensus over the inclusion of benziodiazapine in the guide, although benziodiazapines are not indicated for depression but for anxiety. Whilst this section of the guide is about depression, in pragmatic terms, due to the high volume of anxiety patients seen in China [24] and primary care doctors’ need to make decisions about patients who present both with depression and anxiety concomitantly [82], it seems appropriate that information about Benziodiazapines be provided in this section of the guide.

### Acceptance of person-centered guidelines

This research was unique amongst contextualization studies in this field by testing end-user views on the inclusion of person-centered items in mhGAP-IG.v2 for use in primary care practice. According to the WHO framework for people-centred integrated care*: “All people have equal access to quality health services that are coproduced in a way that meets their life course needs and respects social preferences, are coordinated across the continuum of care, and are comprehensive, safe, effective, timely, efficient and acceptable; and all carers are motivated, skilled and operate in a supportive environmen*t” [83, 84]. Core to this vision is the “empowerment” of the patient, such that a patient’s treatment preferences are respected and their treatment management plan is “co-developed” during a patient-led consultation. Panelists universally embraced the person-centered themes throughout the guide and endorsed the use and development of a *patient management template*. They preferred an action-orientated plan which itemizes patient-centred outcomes in a checklist format rather than the current version which uses more general terms (i.e. “assessment for improvement”; “evaluate engagement and experience in current treatment”). They allocated responsibility for template development to the doctor with a mental health certificate which brings potential benefits of further district-specific contextualization and long-term sustainability of use.

A locally-developed patient management plan would have other advantages too. As part of its strengthening of the mental health system, China is hastening to establish mental health rehabilitation services in the community [85]. Primary care doctors will increasingly work in conjunction, and overlap, with these service providers. Poor communication and coordination between services has been previously identified as a barrier to depression care [30]. Thus the development of a transportable record of follow-up, which is designed to be patient-centred and shared between service providers could ease the transition of care.

### Contextualizing health education

The vast majority of statements put forward to improve psychosocial communications achieved consensus suggesting there is considerable scope to contextualize educational messages. These agreements imply a primary care appetite to provide health information and develop community awareness of depression but highlight a need for direction, grounded by local models of engagement (i.e. previous success stories) on how to achieve these goals. Many of the items correspond to the development of professional “soft skills” (e.g. listening and empathy), which, despite evidence of their positive effect on treatment outcomes [86–88], are often under-addressed in national medical school training programs [89]. A recent review, which identified complex and context-specific communication interactions between health professionals and patients in Asia (including China) also concluded that messaging must move beyond western-based approaches to develop culturally appropriate practices [90]. In Shenzhen, where patients’ psychoeducation needs are diverse and vary by residential district, health education options will need additional consideration.

### Further research

Further development needed at the local level:

(i) Training documents incorporating research findings should be drawn up and shared with local pilot centres for further consultation and testing. A special focus should be given to the development of a patient management plan with a shared use between primary care and multiple partners (including family) involved in patient rehabilitation in the community. Careful consultation with persons with lived experience is recommended to help shape the nature and content of these interactions.
(ii) Development of a training module focusing on soft skills to be incorporated into standard medical training to include a platform of communication aids to address district/demographic differences.
(iii) Qualitative research directed at exploring how primary care can step up activities to educate the community to better recognise depression and improve its treatment response.
(iv) Expanding research to consider the diagnosis and treatment of anxiety and depression comorbid with anxiety.

National information sharing: The findings from this research may not be directly applicable to other cities in China. China is made up of 34 administrative divisions (i.e. 23 provinces, four municipalities, five autonomous regions and two special administrative regions) each with their own distinctive geography and unique population demographics, area-specific disease profile, localised government arrangements and administrative priorities, and mental health and primary health system characteristics. There is also great variation in financial, infrastructure and human resources. Whilst CHCs in Shenzhen are “government owned but hospital managed”, this is not the case in many other Provinces (e.g. Shanghai) where CHCs are both “government owned, and government managed” [91]. Thus, further research is recommended to explore the commonality and differences between CHCs in different cities and to determine how the research findings and/or the method of adaptation could be of value to other potential users of mhGAP-IG.v2. The dissemination of research via publication in relevant medical journals and at local conferences would be an important first step to generating a healthy exchange of information between sites.

Ensure a global feedback-loop: It is important that revised context-specific versions of mhGAP-IG.v2 are published for review by the international body of healthcare researchers. This opens up the feedback loop where other countries can share similar (or different) findings and contribute to the ongoing dialogue on updating global standards of care.

## Limitations

Several limitations of this study are closely related to the COVID-19 pandemic, which spanned the duration of research and directly impacted research feasibility. Wave 1 research coincided with the national call to action for GP panelists to conduct COVID testing and contact tracing of approximately 1 million people who had travelled between Wuhan (the geographic source of the epidemic) and Shenzhen prior to Wuhan’s lockdown [92–95]. Throughout 2020 healthcare services were heavily in demand (to administer care and support vaccine roll-out) and international relations between China and Australia deteriorated [96–99] with the following consequences:

(i) It was not practically feasible, nor ethically appropriate, to initiate the second wave of the survey during much of 2020. This delay, from a pre-planned interval of one month to nearly one year, potentiated a drift in doctors’ perspectives on depression (i.e. confounding) and saw health system changes (e.g. increase in doctor mental health certifications; changes within CHC leadership) which are hard to adjust for in the analysis.
(ii) A loss in panel retention by 21%, although the rate of attrition remained higher than in many panel-based studies conducted in Western countries. Fortunately, the study design over-recruited to allow for a potential loss to follow-up, thus the final number of participants [59] was of sufficient magnitude and included a good spread of panelists from across geographic areas to substantiate findings. This remained well above the recommended minimum panel size (e.g. 10–18) [61] and number for panel stability (e.g. 23) [60] for a Delphi Study.
(iii) A pragmatic decision was taken to terminate the study after two waves of on-line survey with the result that 21% of items remained without further consensus. Whilst many Delphi studies continue to a third wave to fulfil the methodological principle of consensus testing [63], it is not unusual to conduct an adapted, two-wave Delphi study in the mental health research sector [100, 101] due to the cost to benefit ratio of successive waves of research. In this case, two waves of research produced only a tiny change in consensus (1%) and it could be argued that any further research would generate smaller or near negligible changes in viewpoint. Additionally, the body of information produced by the 76% of statements achieving consensus provides abundant, specific and detailed direction for the commencement of guideline adaptation.

This research was designed to obtain the perspectives of primary care physicians only. However, depression care cannot be provided in isolation and further development of the guide would ideally seek advice from multiple stakeholders including the specialist team that receive the referrals, the local services which strive to rehabilitate patients back into the community and, most importantly, persons who live with the day-to-day reality of the condition.

Finally, translation of some concepts/items from the source workshops may have been difficult for primary care doctors to understand. To minimise translation concerns, however, all items were back-translated by an experienced translator and tested prior to fieldwork by two bilingual doctors, working in CHCs in Shenzhen.

## Conclusion

This research asserts that adaptation to mhGAP-IG.v2 is needed in order to align the content and algorithmic flow of decision making for CHC doctors in Shenzhen, China. Panelists gave their consent to diverge from the progenitor guide to accommodate for differences in the context-specific presentation of depression, health system organisation, primary care treatment prioritisation and access, as well as China’s overall person-centered aspirations for twenty-first century care. Uniquely, they assert their preference for a guide that reflects the life course of disease and one that documents the scope of the wider mental healthcare team and family involvement. Equally panelists chose to adopt key measures already contained within the guide for the diagnosis and treatment of bipolar patients, which are outside their current standards of practice. The updating of mhGAP-IG.v2 for the Shenzhen context provides not only a contemporary training tool of local relevance for community healthcare centres but also offers other nations an additional resource to consider in the global advancement of improved treatment and care for depression.

## Supplementary Information

**Additional file 1: Table S3**. Statements achieving consensus. **Table S4**. Statements without consensus.

## Data Availability

Data is stored at the University of Melbourne. The data cannot be freely used, as the study is part of a PhD thesis, with the candidate currently working on the remaining data.

## Abbreviations

MNS: Mental, neurological and substance use disorders
MhGAP-IG.v2: Mental Health Gap Intervention Guide (version 2)
CHC: Community healthcare centre
SSRIs: Selective serotonin reuptake inhibitors
