# Supplementary material for: Adapting the depression component of WHO Mental Health Gap Intervention Guide (mhGAP-IG.v2) for primary care in Shenzhen, China: a DELPHI study
Source: Int J Ment Health Syst. 2022 Feb 15;16:13. doi: 10.1186/s13033-022-00523-0 (PMC8845283; doi:10.1186/s13033-022-00523-0)
Supplement: Supplementary file 1 — Additional file 1: Table S3. Statements achieving consensus. Table S4. Statements without consensus. [file 13033_2022_523_MOESM1_ESM.docx]

#### Supplementary Table 3: Statements achieving consensus. Rank ordered by mean & adjusted mean score (High to Low)

| **Domain 1:- Developing a Patient Centred Guide**  *(Items = 18); % of domain with consensus: 100% (NB 100% of Domain Items achieved Consensus reached in Wave 1)* | **Mean Score** |
| --- | --- |
| 1.07) A patient's risk for suicide should be considered | 4.88 |
| 1.01) Patient privacy should be respected at all times | 4.85 |
| 1.03) It should be not just "patient-centred" but also "person-centred" | 4.81 |
| 1.04) It should include special considerations for specific patient populations | 4.80 |
| 1.09) The management plan may include several strategies to elicit positive behaviour change | 4.71 |
| 1.15) The management plan should emphasize active evaluation of the patient's engagement in therapy | 4.69 |
| 1.14) The management plan should encourage doctors to receive patient's feedback on their treatment experience | 4.68 |
| 1.06) A person's relationship status should be considered | 4.67 |
| 1.05) It should include some examples/case histories of people living in Shenzhen suffering from depression | 4.65 |
| 1.13) The management plan should include agreed dates for return consultations with the CHC Doctor | 4.65 |
| 1.17) The management plan should include regular reviews of any side effects to drug treatment | 4.65 |
| 1.16) The management plan should seek to monitor drug treatment changes | 4.63 |
| 1.10) The management plan must be flexible to evolve over time to suit the patient needs | 4.60 |
| 1.11) The management plan should be developed in partnership with the patient | 4.60 |
| 1.12) The management plan should itemise a patient's individual referral options | 4.56 |
| 1.02) It should provide advice about maintaining family contact during all stages of care | 4.55 |
| 1.08) CHC doctors should provide each patient with a personalized management plan | 4.45 |
| 1.18) Templates for a management plan should be developed by the CHC-doctor in charge of mental health | 4.21 |
| **Domain 2:- Symptom Presentation of Depression**  *(Items = 28); % of domain with consensus: 64%* |  |
| 2.05) Consider previous history of depression early on in consultation. | 4.71 |
| 2.04) Consider patient's suicide risk early in consultation | 4.57 |
| 2.03) Any relationship concerns (including loss of a partner or family member) should be considered early in consultation | 4.49 |
| 2.12) Clarify the number of symptom required for a diagnosis | 4.48 |
| 2.24) Additional symptom information is required for Children | 4.47 |
| 2.06) Flow chart separated into physical and mental symptoms | 4.45 |
| 2.11) Provide symptom profile changes as the disease becomes more severe | 4.44 |
| 2.02) Start assessment with consideration of physical problems first | 4.41 |
| 2.01) Reflect the nature of a "real life" CHC consultation | 4.40 |
| 2.14) Include triggers and risk factors for depression | 4.40 |
| 2.17) Good predictors of depression should be higher in the list | 4.40 |
| 2.15) List more commonly seen symptoms higher-up in Box 1.2 | 4.31 |
| 2.27) Quantify sleeping too much Box 1.2 | 4.25 |
| 2.08) Consider depression as "a spectrum of symptoms" rather than a diagnosis based on Core Symptoms (Box1.1) | 4.24 |
| 2.10) Provide a full symptom listing by merging Box 1.1 and Box 1.2 | 4.23 |
| 2.28) Carefully translate "Talking or moving more slowly than usual" (Box 2.4) | 4.17 |
| 2.13) Symptoms best considered and represented as part of a spectrum | 4.11 |
| 2.22) Include loss of libido in the additional symptom list (Box 1.2) | 4.04 |
| **Domain 3:- Improving Access to and Usage of Tools to Assist Doctors with Depression Diagnoses**  *(Items= 8); % of domain with consensus: 73%* |  |
| 3.10) Dr training needed to conduct depression screeners/diagnostic interviews | 4.73 |
| 3.02) Recommendation for a PHP depression screener required | 4.68 |
| 3.07) Conduct screening in a private consultation room | 4.67 |
| 3.03) Recommend depression screener usage | 4.6 |
| 3.01) Indicate when to use screeners/questionnaires | 4.53 |
| 3.04) Depression screener usage could be introduced into DEP 2 >> Management section | 4.42* |
| 3.11) Depression screener could be conducted by an appropriately trained practice nurse | 4.17 |
| 3.05) Depression screener used in follow-up (DEP 3) | 4.08 |
| **Domain 4:- Healthcare Interconnectivity**  *(Items = 23); % of domain with consensus: 82%* |  |
| 4.27) Highlight community monitoring for suicide risk patients | 4.72 |
| 4.02) Involve professional medical institutions (both public and private), community members and families. | 4.61 |
| 4.03) Partnership with schools to support student mental healthcare | 4.61 |
| 4.01) Identify the roles and responsibilities of CHC and other health workers | 4.59 |
| 4.06) Provide referral and treatment options according to disease severity | 4.59 |
| 4.07) Recommend when to refer to a hospital specialist | 4.59 |
| 4.20) Recognize family and how to engage them in patient care | 4.57 |
| 4.05) Itemise all referral options (both public and private) | 4.45 |
| 4.23) Include:- "Develop, in partnership with your patient, a management plan" | 4.43 |
| 4.09) Include: “Consider referral to hospital experts for psychotherapy” | 4.40 |
| 4.18) Access work-station support groups (where available) | 4.40 |
| 4.21) Provide examples of voluntary sector supporting depression care | 4.36 |
| 4.24) Clarify conditions for police to be informed of a mental health diagnosis | 4.33 |
| 4.17) Highlight the mental health support services available in the community | 4.32 |
| 4.25) "Conduct home visits in the company of a community aid/social worker" | 4.31 |
| 4.26) Guidance on when to conduct home visits with the police | 4.31 |
| 4.08) Provide details of district hospitals as referal alternative to Kangning Hospital | 4.29 |
| 4.28) Clarify doctor role with modifying drug treatment for returning patients | 4.28 |
| 4.11) Indicate when to refer to CHC dr with a mental health certificate | 4.23 |
| 4.13) It should encourage doctors to refer to known/reputable psychiatrists who can be consulted privately | 4.19* |
| 4.16) Include how to involve support staff in the assessment process | 4.17 |
| 4.19) Draw-upon resources at the Disabled Persons Federation | 4.16 |
| 4.10) Include - "Consider referral to hospital experts for antidepressant treatment" | 4.03 |
| **Domain 5:- Considering Pharmacological Interventions for Depression Patients**  *(Items=14); % of domain with consensus 78% (NB No new items added after wave 2)* |  |
| 5.07) Be aware of SSRIs treatment regimen | 4.63 |
| 5.06) Be aware of TCA treatment regimen | 4.57 |
| 5.14) Detail drug indications by depression severity (Table 1) | 4.53 |
| 5.18) Highlight common side effects experienced | 4.53 |
| 5.16) Highlight drugs with slow treatment action and provide treatment adherence advice | 4.52 |
| 5.04) Highlight relative doctor/specialist role in monitoring treatment | 4.41 |
| 5.11) Update to include new generation drugs | 4.39 |
| 5.05) Highlight relative doctor/specialist role in changing the dosage | 4.37 |
| 5.03) Indicate appropriate stage to initiate antidepressants | 4.33 |
| 5.12) Expand to include Benzodiazepine information | 4.29 |
| 5.10) Update with Chinese Brand names | 4.28 |
| 5.15) Include Escitalopram details | 4.28 |
| 5.13) Provide reimbursement details | 4.11 |
| 5.09) Provide drug availability at CHC/Hospital | 4.07 |
| **Domain 6:- Considering Mania and the Potential of Depressive Episode in Bipolar Disorder**  *(Items=4); % of domain with consensus:100% (NB 100% Consensus achieved W1)* |  |
| 6.02) Don't prescribe antidepressants without a mood stabiliser for bipolar disorder (to avoid a manic episode) | 4.64 |
| 6.03) Ask patient's family members about potential symptoms of mania | 4.60 |
| 6.01) People with a depressive episode of bipolar disorder are at risk of mania | 4.59 |
| 6.04) Suggest the duration for monitoring antidepressant + mood stabiliser (e.g. 14 days) | 4.53 |
| **Domain 7:- Communicating Depression to Patients and the Community**  *(Items = 26); % of domain with consensus: 90% (NB No new Items added)* |  |
| 7.25) More strongly emphasise the role of family | 4.64 |
| 7.27) Encourage patients to find pleasure from even little things | 4.61 |
| 7.02) Provide strategies to improve depression awareness in the community | 4.59 |
| 7.20) Highlight the importance of treatment compliance | 4.57 |
| 7.26) Include simple information e.g. encourage the patient to eat and drink properly | 4.57 |
| 7.06) Include drug treatment compliance advice | 4.56 |
| 7.04) Include strategies on how CHC can monitor suicide risk patients | 4.55 |
| 7.23) Encourage patient-return after specialist appointment | 4.55 |
| 7.24) Prepare the patient for the community/society's response to their diagnosis | 4.53 |
| 7.12) Highlight the importance of developing patient trust | 4.52 |
| 7.13) Drs should "listen with empathy" | 4.52 |
| 7.03) Include information on setting-up/managing patient groups | 4.51 |
| 7.09) Supplement Brief psychological treatments with fact sheets | 4.51 |
| 7.21) Provide the patient with strategies to navigate the healthcare system | 4.51 |
| 7.01) CHC must improve the mental health literacy of their resident population | 4.43 |
| 7.22) Highlight the importance of patients attending appointments with specialists | 4.40 |
| 7.08) Encourage doctors to implement an appointment system | 4.39 |
| 7.05) Include strategies for attendance of specialist appointments | 4.37 |
| 7.14) Provide contextually relevant strategies to communicate a depression diagnosis | 4.37 |
| 7.19) Stipulate that Drs and patients agree a healthcare plan | 4.33 |
| 7.15) "Euphemistic talk is better than providing a formal diagnosis" | 4.31 |
| 7.18) Drs should tackle any misconceptions about the disease | 4.31 |
| 7.07) Provide the treatment success stories | 4.28 |
| 7.29) Information about photo-therapy to re-engage in activities | 4.17 |
| 7.11)Drs must play an active role in the psychoeducation of their patients | 4.12 |
| 7.28) Inform how Drs can use Wechat to re-engage patient with activities | 4.07 |
| **Domain 8:- Follow-up**  *(Items = 24) % of domain with consensus 73%* |  |
| 8.04) Assess for improvement | 4.51 |
| 8.03) Determine a patient's current treatment status and establish previous therapy or treatments received | 4.48 |
| 8.02) Outline the importance of patient follow-up | 4.45 |
| 8.09) Monitor the safety-side effect profile of treatment | 4.45 |
| 8.05) Provide basic psychosocial education | 4.44 |
| 8.12) Itemise "signs of improvement" and how to establish these | 4.43 |
| 8.13) Reflect the key modes of follow-up | 4.41 |
| 8.06) Refer patients onto services which provide psychological interventions | 4.40 |
| 8.21) Include advice for non-attendance of referral appointments | 4.40 |
| 8.15) All patients with serious depression should be seen face-to-face | 4.39* |
| 8.28) Contact frequencies should depend on the level of depression severity | 4.37 |
| 8.07) Refer patients onto specialists for drug treatment | 4.33 |
| 8.24) Follow-up should be continuous | 4.33 |
| 8.19) Follow-up must be conducted privately to avoid the patient being the subject of discrimination | 4.32 |
| 8.22) Include the matters to consider to help determine the contact frequency | 4.32 |
| 8.16) Face-to-face contact is the most important follow-up approach and should be encouraged at all times, for all patients | 4.31* |
| 8.14) Stipulate which mode of patient-contact should be initiated under which conditions | 4.21 |
| 8.32) Compliant &depressive symptoms reduced, follow-up extended to every second month | 4.16 |
| 8.17) For non-emergency cases, approach determined according to the patient-preference | 4.13 |
| 8.20) Schedule longer consultation times for follow-up | 4.11 |
| 8.33) Frequency and contact approach organised into six levels | 4.04 |
| 8.30) Recommended contact frequency for severe condition should be every 2-7 days | 4.03* |
| 8.23) The guide should stipulate how often follow-up should be conducted over a set period of time | 4.00* |
| 8.27) Contact frequency is dependent on patient tendency to self harm | 4.00 |
| **Domain 9:- Managing Patient Information**  *(Items = 9); % of domain with consensus: 82% (NB no new statements added)* |  |
| 9.02) Update patient records after each consultation | 4.52 |
| 9.04) Check multiple sources of information relating to the patients condition when providing on-going care | 4.47 |
| 9.05) Validate patient hospitalisation/medication records with the patient's family members | 4.44 |
| 9.01) Utilise the patient management system to review patient history before consultation | 4.41 |
| 9.09) Discuss patient cases directly with specialists and other treating doctors to develop a treatment plan | 4.23 |
| 9.06) Speak directly with specialists/other doctors who have seen the patient to update the patient's records | 4.19 |
| 9.08) Ask patient to bring prescription drugs to consultation | 4.16 |
| 9.10) Hold regular meetings (e.g. once a month) with other doctor(s) based at CHC to discuss difficult cases | 4.15 |
| 9.03) The patient management system does not provide sufficient information about the patient condition. | 4.11 |
| **Domain 10:- Overall Guide Structure**  *(Items = 14;) % of domain with consensus: 74% (NB No new statements added)* |  |
| 10.19) Personally use an adapted guide to improve my understanding of how to assess, manage and follow-up depression patients. | 4.44 |
| 10.06) Include some empty boxes for district-specific information | 4.37 |
| 10.08) Restructure to highlight the difference between depression and a depressive episode in Bipolar disorder | 4.33 |
| 10.05) Less wordy and use more icons | 4.27 |
| 10.10) Restructure to show the management/treatment responsibilities of different members of the healthcare team | 4.25 |
| 10.17) An adapted guide could be used to support changes in mental health policy | 4.24 |
| 10.02) Some restructuring in parts to reflect user context | 4.23 |
| 10.14) Integrate Follow-up into Management section as a continuous process | 4.21 |
| 10.09) Assessment to provide two routes down to show activation of doctor network in parallel to family/community network | 4.20 |
| 10.16) An adapted guide is best used as a training aid | 4.20 |
| 10.01) Major restructure throughout to reflect user context | 4.16 |
| 10.07) Commence assessment by considering patients at high risk of suicide | 4.15 |
| 10.04) Restructure with patient at the centre of each stage of care | 4.01 |
| 10.15) An adapted guide is best used at the point of care | 4.01 |
|  |  |
| * Adjusted Mean Score derived from Wave 2 Research |  |

***Supplementary Table 4: Statements without consensus. Rank ordered by mean & adjusted mean score (Low to High)***

| **Domain 2: - Symptom Presentation of Depression**  *(Items = 10) Non Consensus: 35%* | **Adjusted Mean Score** |
| --- | --- |
| 2.07) The common presentations (Box 1.0) can be deleted from this section | 2.42 |
| 2.09) Patients are more likely to be identified with depression by considering additional symptoms (Box1.2) independently of the core symptoms (Box1.1) | 3.08 |
| 2.25) Remove Physical restlessness from Box 1.2 - as not considered culturally relevant | 3.29 |
| 2.20) Rare symptoms should be included in the additional symptom list (Box 1.2) | 3.39 |
| 2.26) Remove Indecisiveness from Box 1.2 as not considered culturally relevant | 3.47 |
| 2.19) Anxiety should be included in the additional symptom list (Box 1.2) | 3.64 |
| 2.18) Weeping should be included in the additional symptom list (Box 1.2) | 3.71 |
| 2.21) Feeling lonely should be included in the additional symptom list (Box1.2) | 3.75 |
| 2.23) Non-specific, somatic symptoms should be included in the additional symptom list (Box 1.2) | 3.81 |
| 2.16) The list order should place severe symptoms (e.g. suicide) first | 3.93 |
| **Domain 3:- Improving Access to and Usage of Tools to Assist Doctors with Depression Diagnoses**  *(Items = 3) Non Consensus: 27%* |  |
| 3.09) Under certain situations, it is appropriate for a depression screener to be conducted in the police station. | 3.03 |
| 3.08) It is appropriate to conduct a depression screener in the patients home (in the company of a social worker) | 3.76 |
| 3.06) The depression screener should be administered several times during the course of follow-up | 3.97 |
| **Domain 4:- Healthcare Interconnectivity**  *(Items = 5) Non Consensus: 18%* |  |
| 4.04) If so, it should encourage doctors to refer patients to private counsellors in the community (i.e. patients must pay for themselves) | 3.41 |
| 4.14) It should include: - "Consider use of TCM to alleviate symptoms of depression | 3.49 |
| 4.15) It should include: - "Consider acupuncture to alleviate symptoms of depression | 3.63 |
| 4.04) It should emphasize that the community health service centre doctors are mainly responsible for lifestyle adjustments | 3.81 |
| 4.22) It should state: - "Schedule an extended appointment with the patient (when the clinic is less busy) for the purposes of psychoeducation” | 3.95 |
| **Domain 5:- Considering Pharmacological Interventions for Depression Patients**  *(Items = 4) Non Consensus: 22%* |  |
| 5.01) CHC doctors should be responsible for initiating drug treatment for mild and moderate depression patients | 3.29 |
| 5.08) The drugs listed in Table 1 are not standardly available from CHC | 3.36 |
| 5.17) Table 1 should include details of widely used TCM practices for the management of symptomatic relief of depression? | 3.42 |
| 5.02) It should highlight that anti-depressant drug treatment is generally introduced by specialists | 3.95 |
| **Domain 7:- Communicating Depression to Patients and the Community**  *(Items = 3) Non Consensus:10%* |  |
| 7.16) Doctors should communicate to patients that depression is a transitory state | 3.75 |
| 7.17) Doctors should inform patients that depression is a chronic disease | 3.76 |
| 7.10) It should state that it is the CHC doctors‘ responsibility to communicate a depression diagnosis to the patient | 3.81 |
| **Domain 8:- Follow-up**  *(Items = 9) Non Consensus: 27%* |  |
| 8.11) There is no need to make provision for returning patients as their needs are served at hospital | 2.19 |
| 8.01) We successfully follow-up all patients with suspected depression | 2.97 |
| 8.25) The recommended maximum duration for follow-up should be set at 2 months | 3.17 |
| 8.26) The recommended maximum duration for follow-up should be set at 9-12 months | 3.37 |
| 8.31) Recommended contact frequency is dependent to patient compliance | 3.59 |
| 8.10) Follow-up is an opportunity to for CHC doctors to personally modify or change drug treatment regimens | 3.71 |
| 8.29) Recommended contact frequency for mild/moderate condition should be every two-four weeks | 3.76 |
| 8.08) Follow-up is an opportunity for CHC doctors to personally prescribe/initiate anti-depressant treatment | 3.81 |
| 8.18) The choice of follow-up approach should be dependent on Dr time and availability | 3.98 |
| **Domain 9:- Managing Patient Information**  *(Items = 2) Non Consensus: 18%* |  |
| 9.07) Encourage the GP to conduct follow-up at the hospital so that they can personally collect the patient's record of hospitalisation | 3.68 |
| 9.11) GPs to ring patients who have been referred to a specialist (irrespective of whether they receive feedback from the patient management system or not) | 3.78 |
| **Domain 10:- Overall Structure and Use of Adapted Guide**  *(Items =5) Non Consensus: 26%* |  |
| 10.11) In DEP>>2 Management, it is unnecessary to give equal emphasis of both Protocol 1 and 2 | 3 |
| 10.18) An adapted guide has limited practical use for CHC doctors | 3.28 |
| 10.12) DEP>>2 Management should de-emphasise Bipolar Disorder (i.e. Protocol 2) but provide extra safety information about treatment of bipolar disorder | 3.83 |
| 10.03) It should be restructured to put the CHC doctor-activates at the centre of each stage of care | 3.95 |
| 10.13) It is appropriate to have a separate section for DEP>>3 | 3.98 |
